# Supplementary figures and images for: Analysis of motility in multicellular Chlamydomonas reinhardtii evolved under predation
Source: PLoS One. 2018 Jan 30;13(1):e0192184. doi: 10.1371/journal.pone.0192184 (PMC5790280; doi:10.1371/journal.pone.0192184)

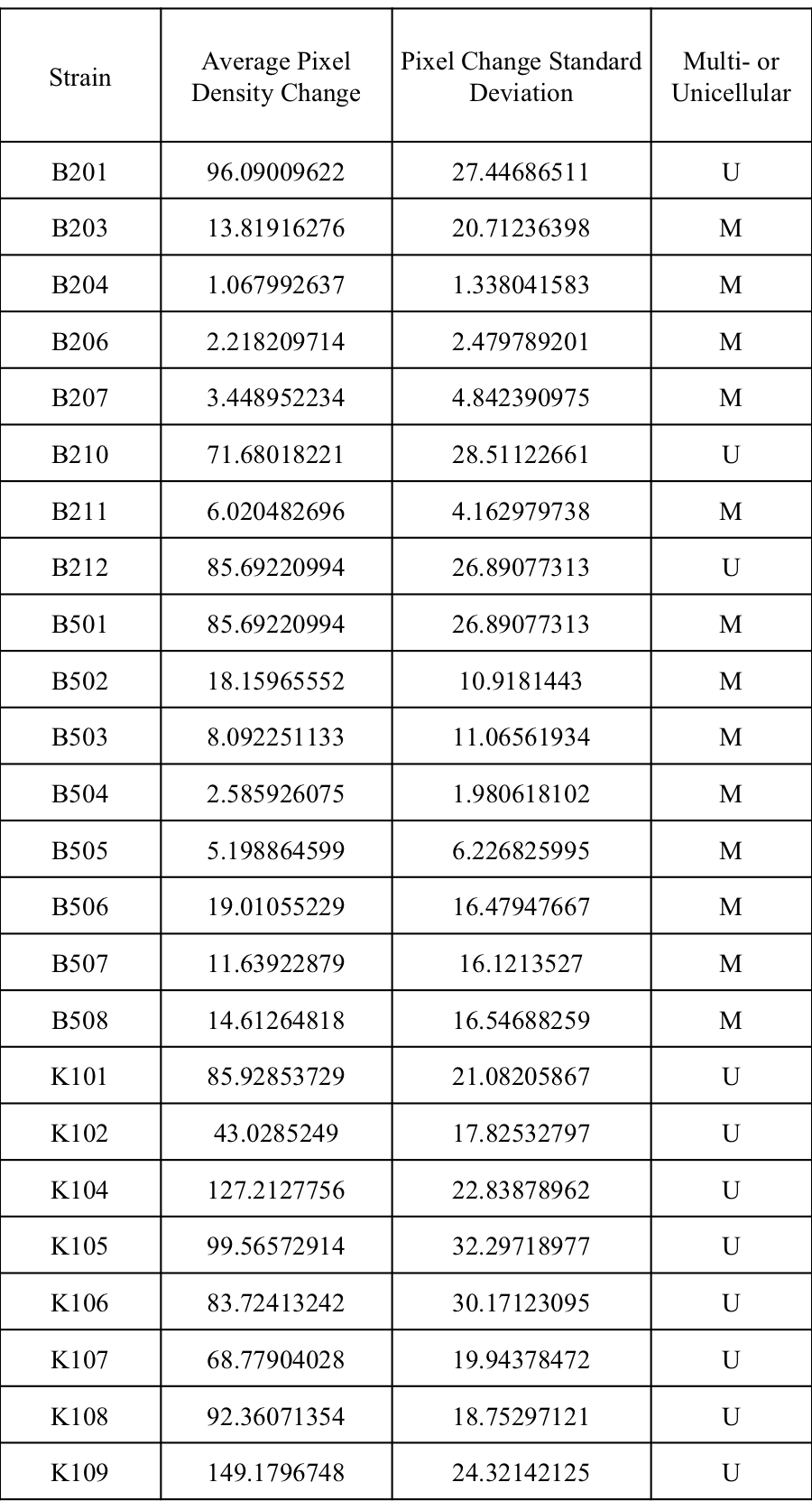

Supplement: S1 Table — Values of the pixel density difference between the front and back half of each well for each strain. (TIFF) [file pone.0192184.s002.tiff]
